# Investigation of nerve fibers in the skin by biopsy: technical aspects, indications, and contribution to diagnosis of small-fiber neuropathy

**DOI:** 10.31744/einstein_journal/2022MD8044

**Published:** 2022-07-04

**Authors:** Irina Raicher, Luís Henrique Casartelli Ravagnani, Silene Gomes Correa, Cristine Dobo, Cristóvão Luis Pitangueira Mangueira, Ricardo Silvestre e Silva Macarenco

**Affiliations:** 1 Hospital Israelita Albert Einstein São Paulo SP Brazil Hospital Israelita Albert Einstein, São Paulo, SP, Brazil.; 2 Universidade Anhembi Morumbi São Paulo SP Brazil Universidade Anhembi Morumbi, São Paulo, SP, Brazil.

**Keywords:** Biopsy, Skin, Nerve fibers, Small fiber neuropathy, Diabetes mellitus, Diabetic neuropathies, Fabry disease, Neuralgia, postherpetic, HIV, Rheumatic diseases, Drug therapy

## Abstract

Skin biopsy with investigation of small-diameter nerve fibers in human epidermis and dermis has been proven to be a useful method for confirming small-fiber neuropathy. In medical practice, small-fiber neuropathy is increasingly recognized as a leading cause of neuropathic pain. It is a prevalent complaint in medical offices, brought by patients often as a “painful burning sensation”. The prevalence of neuropathic pain is high in small-fiber neuropathies of different etiologies, especially in the elderly; 7% of population in this age group present peripheral neuropathy. Pain and paresthesia are symptoms which might cause disability and impair quality of life of patients. The early detection of small-fiber neuropathy can contribute to reducing unhealthy lifestyles, associated to higher incidence of the disease.

## INTRODUCTION

Skin biopsy to investigate small-diameter nerve fibers in the human epidermis and dermis has proved to be a reliable diagnostic tool for confirmation of small-fiber neuropathy. By definition, a pure small-fiber neuropathy cannot be diagnosed by standardized nerve conduction studies, since these only reflect the function of larger, myelinated nerve fibers. A quantitative sensitivity testing may show impairment suggestive of small-fiber neuropathy, but this method is heavily dependent on patient cooperation, and does not differentiate lesions involving peripheral and central sensory pathways. Skin biopsy to investigate small-diameter nerve fibers was widely used during the last two decades in the diagnostic evaluation and follow-up of patients with peripheral neuropathy.^(1-7)^ This article summarizes data on skin biopsy use as a method to investigate nerve fibers and its indications, with emphasis on epidermal nerve fibers.

### Technical aspects

**Figure 1:**
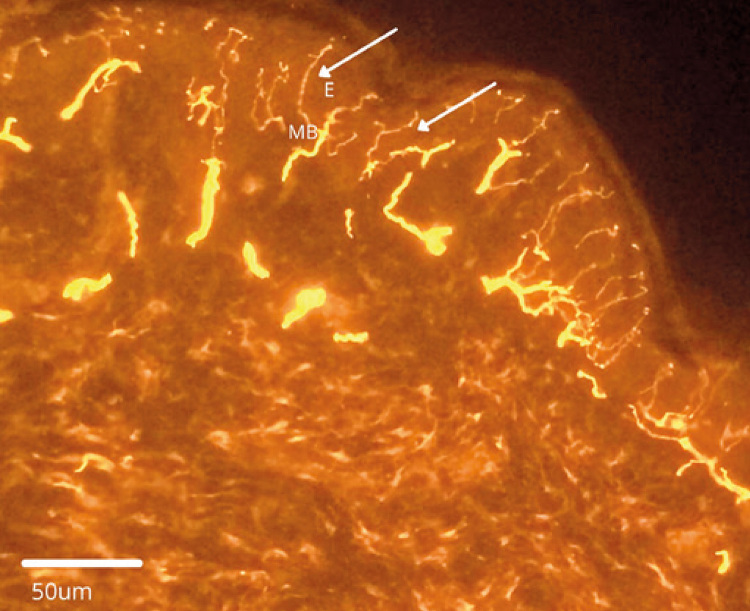
Intraepidermal nerve fibers (arrows) stained with primary antibodies to the pan-axonal marker PGP 9.5 (1:800 Cedarlane®, Ontario, Canada) and Cy3-labeled secondary antibodies (1:100 Jackson®, PA, USA). Immunofluorescence microscopy, 200x magnification

By immunostaining for the protein gene product 9.5 (PGP 9.5), a pan-axonal neuronal marker, nerve bundles are observed as they form horizontal subepidermal neural plexuses in the papillary dermis and traverse vertically through the epidermal basement membrane, ascending between the epithelial cells as intraepidermal nerve fibers. They end superficially as free nerve endings (Figure 1).

E: epidermis; MB: basement membrane.

The intraepidermal nerve fibers are unmyelinated, as they lose their Schwann cell sheathing them at the dermal-epidermal junction.^(7)^

Biopsy is most commonly performed using a 3-to- 5mm disposable circular punch under sterile technique, and after local anesthesia with lidocaine.^(7)^ No sutures are usually needed, and minor complications (excessive bleeding, infection, etc.) are very rare. In most cases, healing occurs within 7-10 days. The biopsy contains epidermis and dermis, including sweat glands.

The technique was first described by researchers at the Karolinska Institute, in Sweden,^(8)^ and later developed and standardized at the University of Minnesota,^(9)^ and at the Johns Hopkins University.^(10)^

In most peripheral neuropathy studies, skin biopsies are obtained from the distal part of the leg (10cm above the lateral malleolus) and, in some cases, also from the upper lateral aspect of the thigh (20cm below the anterior iliac spine).^(7)^ A second sample from a proximal site is chosen to detect the length-dependent cutaneous nerve fiber loss, a typical feature of axonal polyneuropathy.^(7)^

A joint task force of the European Federation of Neurological Societies and the Peripheral Nerve Society,^(7)^ in 2010, established guidelines for the quantification of intraepidermal nerve fibers and recommendations for normative values. Quality control measures are recommended at all stages of processing to ensure those who will read the skin biopsy results have the best conditions to do it. Since laboratory techniques are generally not automated, artifacts and errors can occur in the processes of cutting, staining, or interpreting and counting. Intra- and interobserver measurements and inter-laboratory quality control are recommended periodically.^(7)^

### Indications

A skin biopsy to quantify intraepidermal nerve fibers may be considered for diagnosis in patients with symptoms suggestive of small-fiber neuropathy, and in many patients with neuropathic pain. Considering the degeneration of small nerve fibers cannot be detected by routine electrophysiological tests, this diagnosis is often difficult to establish in clinical practice. A skin biopsy does not reveal, in most cases, the etiology of the small-fiber neuropathy, but this diagnosis often promotes an earlier investigation of its causes, such as diabetes or intolerance to glucose,^(11)^ among others. In some cases, cutaneous vasculitis may be demonstrated.^(12-14)^ Non-length-dependent values of nerve fiber density may also indicate the occurrence of sensory ganglionopathy (paraneoplastic or associated with Sjögren syndrome).^(15)^ In table 1, some studies that show contributions of this test to diagnosis and follow-up of small-fiber neuropathy patients are cited.^(16-48)^

**Table 1:** Contribution of the investigation of nerve fibers in the skin by biopsy in peripheral nerve diseases

Indication	Finding	References
Diabetic neuropathy	Diabetes induces early degeneration of nerve fibers in the skin correlated with the duration and severity of the condition	Kennedy et al.^(16)^ Pittenger et al.^(17)^ Sorensen et al.^(18)^
Diabetic neuropathy	Individuals with diabetes have slower regeneration, even with no evidence of neuropathy	Polydefkis et al.^(19)^
Diabetic neuropathy	Studies from North America and Europe have shown different results on the effect of glucose intolerance on intraepidermal fiber loss	Smith et al.^(11)^ Sumner et al.^(20)^ Løseth et al.^(21)^ Nebuchennykh et al.^(22)^ Vlckova-Moravcova et al.^(23)^
Small-fiber neuropathy	A skin biopsy is the most sensitive method for diagnosing small-fiber neuropathy	Periquet et al.^(24)^ Devigili et al.^(25)^ Scherens et al.^(26)^
Small-fiber neuropathy	The progressive nature of idiopathic small-fiber neuropathy is demonstrated by repeated skin biopsies	Lauria et al.^(27)^
Small-fiber neuropathy	There are length-dependent and non-length-dependent small-fiber neuropathies	Holland et al.^(28)^ Lauria et al.^(29)^ Uçeyler et al.^(30)^
Inflammatory demyelinating neuropathy	Guillain-Barré syndrome and chronic inflammatory demyelinating polyradiculoneuropathy may present with loss of intraepidermal nerve fibers	Chiang et al.^(31)^ Pan et al.^(32)^
Inflammatory demyelinating neuropathy	Anti-MAG neuropathy may show IgM deposits in dermal myelinated nerve fibers	Lombardi et al.^(33)^ Stalder et al.^(34)^
Vasculitic neuropathy	Reduced cutaneous innervation may correlate with the severity of the vasculitic neuropathy, even in the absence of sensory symptoms.	Tseng et al.^(12)^
Vasculitic neuropathy	The presence of skin vasculitis may indicate a non-systemic vasculitic neuropathy with good sensitivity and specificity.	Uçeyler et al.^(13)^
HIV neuropathy	Intraepidermal fiber density is length-dependently reduced in HIV when the patient shows signs of neuropathy regardless of symptoms	Herrmann et al.^(35)^
Hereditary neuropathy	Evaluation of dermal myelinated fibers may show changes in the molecular architecture of the axolemma	Li et al.^(36)^ Sabet et al.^(37)^
Hereditary neuropathy	Dermal myelinated fibers in Charcot-Marie-Tooth 1A have internodal length shortening	Saporta et al.^(38)^
Hereditary neuropathy	Intraepidermal nerve fiber losses in Fabry disease, Friedreich’s ataxia, and familial dysautonomia (Riley-Day syndrome)	Scott et al.^(39)^ Nolano et al.^(40)^ Hilz et al.^(41)^ Torvin Møller et al.^(42)^
Autonomic neuropathy	A peculiar pattern of cutaneous denervation characterizes rare conditions, such as congenital insensitivity to pain with anhidrosis, cold-induced sweating syndrome, and “Ross Syndrome”	Nolano et al.^(40)^ Nolano et al.^(43)^ Di Leo et al.^(44)^
Nerve regeneration	The chemical model with topical capsaicin can be used to study the degenerative and regenerative properties of epidermal nerve fibers	Simone et al.^(45)^ Nolano et al.^(46)^ Nodera et al.^(47)^ Polydefkis et al.^(19)^ Hahn et al.^(48)^

## Contribution to the diagnosis of small-fiber neuropathy

### Diabetic neuropathy

A number of studies have demonstrated small-diameter nerve fibers impairment in skin biopsy specimens from both diabetic patients and experimental diabetic animals. Prospective studies showed diabetes induced early degeneration of skin nerve fibers correlated with the duration of the disease.^(17)^ Intraepidermal nerve fiber densities were reduced in type 2 diabetic patients when compared to age-matched controls, and a correlation has also been shown with increased heat perception thresholds, and reduced sural sensory nerve amplitudes.^(49)^ An inverse association between intraepidermal nerve fiber density and severity of neuropathy has also been demonstrated.^(16,18)^

There is strong evidence of reduced intraepidermal nerve fiber density in patients with glucose intolerance.^(11,20)^

There is an indication that lifestyle interventions can improve nerve fiber density, but this needs to be confirmed in a larger sample.^(50)^ Intraepidermal nerve fiber density is also reduced in patients with glucose intolerance neuropathy with normal nerve conduction recordings.^(11)^ Therefore, quantification of intraepidermal nerve fibers could be used both for early detection of diabetic neuropathy or to assess its progression in trials and in clinical practice.^(51,52)^

### Small-fiber neuropathy

Patients with a clinical diagnosis of idiopathic small-fiber neuropathy often present with painful burning feet. On clinical examination, decreased sensitivity to pain stimuli can be found, whereas proprioception, strength, tendon reflexes and nerve conduction are normal. The skin biopsy usually shows decreased distal intraepidermal nerve fiber density with normal proximal nerve fiber densities, indicating that this condition is typically length dependent.^(28,53)^

Repeated skin biopsies from patients with idiopathic small-fiber sensory neuropathy, with duration of 12 to 28 months, have shown a decrease in the intraepidermal nerve fiber density in the legs and, therefore, a progressive nerve fiber degeneration in this condition.^(27)^ A skin biopsy with determination of intraepidermal nerve fiber density was also considered more sensitive than the quantitative sudomotor axonal reflex test, or the quantitative sensitivity test in the diagnosis of small fiber neuropathy.^(24,26)^

### Sensory ganglionopathy

Patients with sensory ganglion degeneration typically present with sensory ataxia symptoms and loss of conscious proprioception. The distribution of the sensory dysfunction is usually asymmetric and generalized.^(54-56)^ In patients with ganglionopathy, skin biopsies show nerve fiber loss in all sites without the proximal-distal gradient found in distal sensory polyneuropathy.^(29)^

### HIV

HIV infection is a known cause of painful neuropathy with predominant involvement of small-diameter nerve fibers.^(57,58)^ Two different subtypes have been defined: one primarily associated with the HIV infection (HIV-associated distal sensory neuropathy), and the other associated with the drug-specific neurotoxicity of the antiretroviral drugs (toxic antiretroviral neuropathy). Intraepidermal nerve fiber density was reduced in a length-dependent manner in HIV-infected patients with neuropathic signs on physical examination, independently of neuropathic symptoms.^(35)^ Skin biopsies have become part of the diagnostic investigation of patients infected intravenously and was also applied in clinical trials on nerve growth factors.^(59,60)^

### Hereditary neuropathies

Skin biopsy has been studied in patients with hereditary neuropathies known to have small-diameter nerve fiber involvement, such as Fabry disease,^(39,61,42)^ familial dysautonomia,^(41)^ and congenital insensitivity to pain.^(62)^ In these conditions, the depletion of intraepidermal nerve fibers has been demonstrated to be more or less extensive. Similarly, in patients with Friedreich’s ataxia, which initially causes degeneration of the dorsal root ganglia and large-caliber myelinated fibers, skin biopsy revealed involvement of small-diameter nerve fibers in the pathological process.^(40)^

Charcot-Marie-Tooth neuropathy is caused by mutations in myelin proteins, but skin biopsy with evaluation of dermal myelinated nerve fibers showed similar abnormalities in the molecular architecture of the axolemma,^(36,37)^ as previously demonstrated in sural nerve biopsies. Shortening of internodal length was recently described in dermal myelinated fibers of Charcot-Marie-Tooth 1A patients.^(38)^

### Systemic lupus erythematosus

Recent studies have shown small-diameter nerve fibers involvement in biopsy specimens, with decreased intraepidermal nerve fiber densities in systemic lupus erythematosus (SLE).^(63-65)^

Pathogenesis of peripheral nervous system involvement in SLE is poorly understood, and different mechanisms are implicated in length-dependent small-fiber neuropathy,^(63)^ such as immunoglobulin deposition on the nerve surface and activation of the dermal vascular endothelium, leading to a decrease in the fiber density of the intraepidermal unmyelinated nerves.^(63,65)^

A current investigation involving pain symptoms and decreased sensation of warmth in the feet of SLE patients has shown that more than a third of patients require a skin biopsy to diagnose small-fiber neuropathy, since conventional neurological examinations for neuropathy have not been shown to be sufficiently effective.^(64)^ This adds further support to the concept that a pure neuropathy of small diameter nerve fibers can occur in SLE.^(63)^

### Other conditions

Skin biopsy has also been used to demonstrate small-diameter nerve fiber degeneration in a number of conditions other than those mentioned above. In leprosy, cutaneous nerve fibers are typically affected,^(66,67)^ and it has been suggested that quantifying intraepidermal nerve fibers in skin lesions may serve as an additional tool, along with skin smear examination and nerve conduction studies, to increase diagnostic efficacy in the detection of neuropathy in leprosy control programs.^(67)^ Small-fiber neuropathy has been described in patients with hypothyroidism^(68)^ and has also been demonstrated by skin biopsy.^(69)^ In a recently published case report, clinical and neuropathological recovery occurred after replacement therapy.^(70)^

Chemotherapeutic agents produce acute neurotoxicity, chronic neuropathy, and painful neuropathy with skin denervation.^(71)^ Intraepidermal nerve fiber loss has also been reported in patients with Ross syndrome (characterized by the triad of tonic pupils, hyporeflexia, and segmental anhidrosis),^(43)^ erythromelalgia,^(72)^ type 1 complex regional pain syndrome,^(73,74)^ and late-onset restless legs syndrome.^(75,76)^ In restless legs syndrome it has been suggested that hyperactivity of spinal structures in this condition may be triggered by nociceptive deafferentation in a subgroup of patients with painful polyneuropathy.^(77)^ Skin biopsies have also been used in Parkinson’s disease to demonstrate degeneration of autonomic nerves in blood vessels, sweat glands, and erector pili muscles.^(78)^ A neuropathy with predominant small-fiber involvement may be a potential complication of celiac disease. Patients improved with a gluten-free diet.^(79)^

Unexpectedly, in patients with motor neuron disease, it has also been shown that amyotrophic lateral sclerosis (ALS) reduces intraepidermal nerve fiber densities, indicating the presence of a distal and sensory axonopathy in ALS.^(80)^

## CONCLUSION

Skin biopsy for the investigation of intraepidermal nerve fibers is a technique that has made it possible, in a relatively simple way, to visualize small-diameter unmyelinated fibers and, therefore, to evaluate small-fiber neuropathy in a more objective way than the quantitative sensory testing and thermal thresholds.

The test contributes to earlier detection of neuropathy in diseases with dysfunctional glucose metabolism, such as *diabetes mellitus* and glucose intolerance, in connective tissue diseases, paraneoplastic diseases, thyroid disorders, HIV, leprosy, celiac disease, restless legs syndrome, exposure to neurotoxic drugs, hereditary diseases, amyotrophic lateral sclerosis, among other underlying causes of small-fiber neuropathies. Skin biopsy has its role in differentiating between length-dependent and non-length-dependent small-fiber neuropathies, which helps in the differential diagnosis of peripheral neuropathies, narrowing the etiological possibilities, therefore avoiding unnecessary investigation. The causes of ganglionopathy have a smaller differential range (*e.g.*, paraneoplastic ganglionopathy, Sjögren’s syndrome, some viral infections, and chemotherapies) than distal axonopathy, so the pattern aids in a narrower etiologic diagnosis, without, however, determining the causes of the ganglionopathy. The etiological investigation of small-fiber neuropathies with targeted complementary exams is important to allow the identification of treatable diseases. Skin biopsies can also be used to identify small fiber involvement in mixed neuropathies and for follow-up studies.
